# Cardioneuroablation for reflex syncope or functional bradyarrhytmias: new insight from a single center experience

**DOI:** 10.3389/fcvm.2024.1526825

**Published:** 2025-01-13

**Authors:** Noemi Valenti, Antonio Di Monaco, Imma Romanazzi, Nicola Vitulano, Federica Troisi, Federico Quadrini, Antonio Vitullo, Luca Sgarra, Rosa Caruso, Vincenzo Anzelmo, Pietro Guida, Natale Daniele Brunetti, Massimo Grimaldi

**Affiliations:** ^1^ Department of Cardiology, General Regional Hospital “F. Miulli”, Bari, Italy; ^2^ Department of Cardiology, Hospital “San Paolo”, Bari, Italy; ^3^ Department of Medical and Surgical Sciences, Foggia University, Foggia, Italy

**Keywords:** reflex syncope, bradycardia, atrio-ventricular block, cardioneuroablation, neuromodulation, catheter ablation

## Abstract

**Background:**

Cardioneuroablation (CNA) is a new approach to treat reflex syncope and functional bradyarrhytmias caused by autonomic imbalance. We report our experience using CNA.

**Method:**

From September 2022 to July 2023, we took care of 21 patients (mean age 42 ± 21 years; 62% male) affected by reflex syncope or functional bradyarrhythmias. All patients underwent CNA under conscious sedation targeting the superior and/or inferior paraseptal ganglionated plexus (GPs).

**Results:**

Nine patients were affected by vasovagal syncope (VVS) and twelve by functional bradyarrhythmias. In 3 cases (14%) the ablation was performed only on the GPs of the right atrium, while in the remaining 86% of cases we performed biatrial lesions. As regards the acute results, we highlighted an increase in sinus heart rate (12 ± 15 bpm, *p* = 0.001), a shortening of the PQ interval (−18 ± 18 msec, *p* < 0.001), a reduction of the correct sinus node recovery times (cSNRT) (−142 ± 204 msec, *p* = 0.114), a shortening of the AH interval (−31 ± 26 msec, *p* = 0.008), a reduction of the effective refractory period of the atrio-ventricular node (−156; interquartile range from −30 to −160 msec, *p* = 0.042) and an increase in the Wencheback point (27 ± 20 bpm, *p* < 0.001). At follow-up, a single patient, due to persistent symptoms and bradyarrhythmic disorder, underwent permanent pacemaker implantation; no other patient had recurrence of syncope, and all remained persistently asymptomatic.

**Conclusion:**

Our results confirm the efficacy and safety of CNA for the treatment of VVS and functional bradyarrhythmias, although further studies are needed to support these findings.

## Introduction

Cardioneuroablation (CNA) is a new approach to treat reflex cardioinhibitory syncope and functional bradyarrhythmias caused by autonomic imbalance. Catheter-based ablation of the intrinsic cardiac autonomic nervous system, particularly ganglionated plexi embedded in atrial epicardial fat, aims predominantly at parasympathetic denervation. In 2005, Pachon et al. proposed cardiac vagal denervation to treat reflex syncope (1–3). Since then, several studies, including a meta-analysis of retrospective and prospective observational studies, and a recent randomized controlled trial (RCT), have provided some evidence that CNA is able to prevent syncope recurrence at least during the first 2 years following the procedure in patients affected by reflex syncope (2, 4–6). The recent meta-analysis of 14 studies, for a total of 465 patients, reported a mean freedom from syncope recurrence in 92% of patients during follow-up (6). The RCT (ROMAN study) reported a recurrence rate of syncope of 8% in 24 patients randomized to CNA and 54% in 24 controls treated conservatively with optimal nonpharmacological management (*P* = 0.0004) during a 2-year follow-up (5). There is also ample evidence regarding the use of CNA for the treatment of functional bradyarrhythmias, although not derived from randomized studies (3, 7–9).

Although CNA appears to be safe in small study populations with relatively short follow-up in selected patients, experimental studies and prospective clinical observations speculate regarding detrimental consequences of sympathovagal imbalance on cardiac metabolism, inflammation, and electrophysiology (10–12).

In this article, we report our experience in performing CNA in patients with reflex syncope and functional bradyarrhythmias.

## Methods

From September 2022 to July 2023, a total of 21 patients (mean age 42 ± 21 years; 62% male) were referred to our center for reflex syncope or functional bradyarrhythmias. In particular, patients with vasovagal syncope (VVS) were considered for CNA if they had multiple syncopal episodes with evidence of a prevalent cardioinhibitory reflex on tilt testing (VASIS 2B), and after failure of optimal non-pharmacological management (optimization of hydration and salt intake with the diet, adequate physical activity, use of elastic-compressive stockings, counter-pressure maneuvers).

While in patients with VVS bradycardia is limited to the time of the syncopal episodes and the rhythm is normal for the rest of the time, some patients present frequent episodes of sinus bradycardia, asystolic pauses or atrioventricular block (AVB) even in the absence of syncope, but often associated with asthenia, dyspnea, irritability, inability to concentrate, disinterest, forgetfulness, dizziness and fainting, although a clear cause-effect relationship between symptom and bradycardia is often difficult to demonstrate (13). The extrinsic nature of bradyarrhythmia is suspected in case of intermittent episodes, which often occur at rest and during sleep, and then disappear during exercise, frequently in young subjects. These patients were subjected to CNA after demonstration of the existence of a dominant functional component (extrinsic, vagal) underlying the bradyarrhythmic disorder, through disappearance or substantial improvement of the rhythm during ergometric test and/or atropine test.

All patients had a positive response during atropine test (0.04 mg/kg max 2 mg ev). The test was considered positive if heart rate (HR) increased >25% from baseline value or if the AV block disappeared. Furthermore, all patients performed transthoracic echocardiogram, 24 h ECG-Holter monitoring with Heart rate variability evaluation, tilt up test and carotid sinus massage.

All patients were offered the implantation of a permanent pacemaker in accordance with the guidelines of the European Society of Cardiology (14), which they refused. All patients then performed CNA as previously described. All procedures were performed by expert operators (NV, ADM, MG). The study was approved by the local Ethical Review Authority and conducted in full compliance with the Declaration of Helsinki. Informed consent was provided by all patients who were consecutively enrolled.

### Cardioneuroablation

All procedures were carried out in conscious sedation with intravenous infusion of diazepam (max 10 mg) paracetamol (1 g) and fentanyl (0.05–0.2 mg). Ultrasound (US)-guided right femoral vein puncture was performed in all patients. The mapping catheter Pentaray (Biosense Webster, Diamond Bar, USA) was advanced into the right atrium through the femoral sheat. The right atrial anatomy was assessed using the CARTO system (Biosense Webster, Diamond Bar, USA). An electrophysiological study was performed recording sinus node recovery time (TRNS), effective refractory period (ERP) of the atrio-ventricular node, Wenckebach point (PW), AH interval, HV interval, His duration. The activation mapping in sinus rhythm was used to localize the early right atrial activation (sinus node area). Moreover, high output pacing was used to localize right phrenic nerve course. The anatomical approach was based on the presumed anatomical location of the paraganglia. Alternatively, a high frequency stimulation (HFS) was used to localize the superior and inferior paraseptal ganglionated plexus (PSGP). In particular, we performed in the right or left atrium a HFS (20 Hz, 10 V, 10 ms pulse width) for 2–5 s to triggering a vagal response in the form of marked bradycardia (cardiac stimulator: Qubic Stim – Biotronik Inc., USA) (10).

The ablation catheter QDOT (Biosense Webster, Diamond Bar, USA) was used in all patients and was initially inserted into the right atrium. In order to perform sinus node CNA, radiofrequency was delivered at the superior PSGP using this ablation setting: contact force from 10 to 20 g, power 30–40 W, Ablation Index 550. In order to perform AV node CNA, radiofrequency was delivered at the inferior PSGP using this ablation setting: the lesions at the interatrial septum were performed using the contact force from 10 to 20 g, power 30–40 W, Ablation Index 550; the lesions on the posterior wall of the coronary sinus ostium were performed using the contact force between 5 and 20 g, power 28 W, ablation index 350.

If satisfactory electrophysiological parameters were not achieved, CNA was performed in the left atrium, particularly at the level of the left side of the superior and inferior PSGP, and, if still insufficient, at the level of the left superior ganglion plexus (LSGP).

Transseptal access to the left atrium was achieved as previously described (15). Preceding left atrial access, a bolus of heparin (50 U/Kg) was intravenously administered. Following transseptal puncture, heparin boli were intravenously administered up to ACT levels >300 s. The trans-septal sheath was continuously irrigated with heparinized saline (2 ml/min) by a Cool Flow Pump (Biosense Webster, Diamond Bar, USA). The ablation catheter was advanced into the left atrium through the trans-septal sheath. The ablation setting in the left atrium was contact force from 10 to 20 g, power 30–40 W, Ablation Index 550 msec. A complete electrophysiological study was repeated after the ablation.

Procedural endpoints of CNA are yet to be determined. Based on the literature data, depending on the patient's clinical disorder, we used as indicators of procedural success the increase of sinus heart rate (>25%), the reduction of correct sinus node recovery time (cSNRT), the shortening of PQ interval (>25%), the reduction of AH interval and AV node ERP, the increase of PW. For patients with persistent AVB, we aimed to obtain a stable 1:1 atrioventricular conduction. During the procedure, obtaining the same improvement previously recorded after atropine injection, was considered a satisfactory goal.

### Clinical follow up

All patients repeated the day after procedure and after 12 months of follow-up a head-up tilt test and a 24 h ECG-Holter monitoring with HRV. We performed a monthly telephone follow-up to evaluate the patients' symptoms. We evaluated also the increase in minimum-average-maximum heart rate, shortening of the PQ interval, changes in QTc interval, reduction of time and frequency domain parameters in the analysis of heart rate variability (HRV) on ECG Holter monitoring.

### Head-up tilt test

The head-up tilt test (HUTT) in useful in order to assess the autonomic substrate for vasovagal syncope (VVS). During the test, performed under continuous blood pressure and electrocardiographic monitoring, after a short control phase in the supine position, patients are raised to an upright position at 70° for 20 min (passive phase). In case of a negative result, a nitrate (0.4 mg sublingually) is administered, and the tilt is maintained for up to 20 min after drug administration. A positive response is defined as pre-syncope or syncope, clinically compatible with home symptoms, associated with hypotension and/or bradycardia. The VASIS classification according to the response to the HUTT distinguishes between syncope of: type 1 (mixed response with hypotension and bradycardia: heat of blood pressure followed by a drop in heart rate that produces syncope, without reaching 40 bpm); type 2A (cardioinhibitory without asystole: drop in heart rate <40 bpm for >10 s); type 2B (cardioinhibitory with asystole >3 s); type 3 (pure vasodepressor response: heat of blood pressure with increase in heart rate) (16).

### Heart rate variability

Heart rate variability (HRV) derived from an analysis of the RR interval of the ECG is a non-invasive method to assess the function of the autonomic nervous system (17–19). High levels of HRV indices are generally a sign of efficient autonomic mechanisms that characterize a healthy individual, while low HRV often indicates a malfunction of the autonomic nervous system that may be related to various cardiac pathologies (19). In particular, it can be stated that vagal activation is associated with an increase in HRV, while sympathetic activation is associated with a reduction in HRV (20, 21). HRV analysis is usually based on long-term Holter ECG recordings (at least 18 h) or short-term ECG recordings (usually 5 min), and can be performed with different methods, including time and frequency domain analyses, as well as nonlinear techniques (20). Time domain variables depend on the simple measurement of R-R intervals, to then obtain, through simple mathematical techniques, statistical and geometric indices, with results expressed in units of time (milliseconds) (19, 21). Among the time domain parameters, the most widely used in literature are the mean heart rate (HR), the SDNN (standard deviation of all intervals from normal to normal) and the RMSSD (root mean square of the differences between successive normal heart beats), as well as the pNN50 (percentage of differences between consecutive RR intervals >50 msec) (6, 21). Frequency domain variables express the amplitude (or power) of the oscillations of the RR intervals in four specific frequency cycles: ultra-low frequency (ULF 0–0.003 Hz), very-low frequency (VLF 0.003–0.04 Hz), low frequency (LF 0.04–0.15 Hz), and high frequency (HF 0.15–0.40 Hz). HF depend mainly on vagal activity; LF are representative of sympathetic and vagal modulation, with sympathetic predominance; VLF and ULF have a more complex and multifactorial genesis (19, 21). The global sympathovagal balance can be represented by the ratio between the LF and HF components: in healthy adults, the LF/HF ratio during rest is 1:2 (22).

### Statistical analysis

Based on the descriptive nature of study, no formal sample size calculation was performed. Summary statistics were reported as mean ± standard deviation or median with interquartile range for continuous variables and number of subjects, frequency or percentage for categorical data. Difference in a continuous variable between the two time points was evaluated by paired samples Student's *t*-test or Wilcoxon matched-pairs signed-rank test when appropriate. The Shapiro-Wilk test was used to check deviation from normal distribution. A *p*-value of 0.05 or less was considered statistically significant. All analyses were performed using STATA version 16 (StataCorp, College Station, Texas).

## Results

### Basic characteristics of the study population

The main clinical characteristics of patients are reported in Table 1. In particular, 8 patients were affected by vaso-vagal syncope (VVS) of cardioinhibitory type (VASIS 2B) documented by an HUTT, 1 patient had a mixed type VVS (VASIS 1) and contextual documentation of asystolic pauses by a previously implanted loop recorder, the other 12 presented functional bradyarrhythmias (4 cases of marked sinus bradycardia, 8 cases of episodic or fixed high-grade atrio-ventricular block; in particular, a 19-year-old patient on whom we recently published a clinical case, presented fixed third-degree atrioventricular block with an average ventricular rate of 41 bpm) (23). All patients were symptomatic due to recurrent syncope (100%), lipothymia (33%), dyspnea (14%), asthenia (38%), dizziness (5%), palpitations (14%). The syncopal episodes in some cases caused significant trauma (3 episodes of head trauma) or were dangerous because they occurred unexpectedly even while driving or while carrying out work. In particular, the 4 patients with marked sinus bradycardia were categorized in the functional bradyarrhythmia group because, although they had experienced syncopal episodes, they mainly complained of frequent episodes of marked sinus bradycardia or asystolic pauses not necessarily associated with syncope.

**Table 1:** Study population.

Population's characteristics	*n* = 21
Female sex	8 (38%)
Age	42 ± 21
Vaso-vagal syncope	9 (43%)
Sinus bradycardia	4 (19%)
Atrio-ventricular block	8 (38%)
Structural heart disease	1 (5%)
Syncope	21 (100%)
Lipothymia	7 (33%)
Dyspnea	3 (14%)
Asthenia	8 (38%)
Dizziness	1 (5%)
Palpitations	3 (14%)

### Procedural aspects

The procedural aspects are reported in Table 2. Mean procedure and fluoroscopy times were 118 and 2,3 min, respectively. In 11 cases (52%) the procedure was performed without fluoroscopy, with the aid of the electroanatomic mapping system. In 6 cases (29%) the CNA was guided by high-frequency stimulation while in the other 15 cases (71%) it was performed with anatomical approach.

**Table 2:** Procedural aspects.

Procedural aspects	*n* = 21
Anatomical CNA	15 (71%)
CNA driven by HFS	6 (29%)
Right CNA	3 (14%)
Biatrial CNA	17 (86%)
GPs for sinus node only	3 (14%)
GPs for atrio-ventricular node only	8 (38%)
GPs for both sinus and atrio-ventricular node	10 (48%)
SPSGP	12 (57%)
IPSGP	18 (86%)
LSGP	9 (43%)

In 3 cases (14%) the ablation was performed only on the ganglionated plexi (GPs) of the right atrium, while in the remaining 86% of cases we performed biatrial lesions. In particular, in 14% of cases the ablation affected only the GPs that mainly innervate the sinus node (SPSGP and LSGP), in 38% of cases only the GPs that mainly innervate the AV node (IPSGP), in 48% of cases of cases both (SPSGP, LSGP, IPSGP).

**Figure 1:**
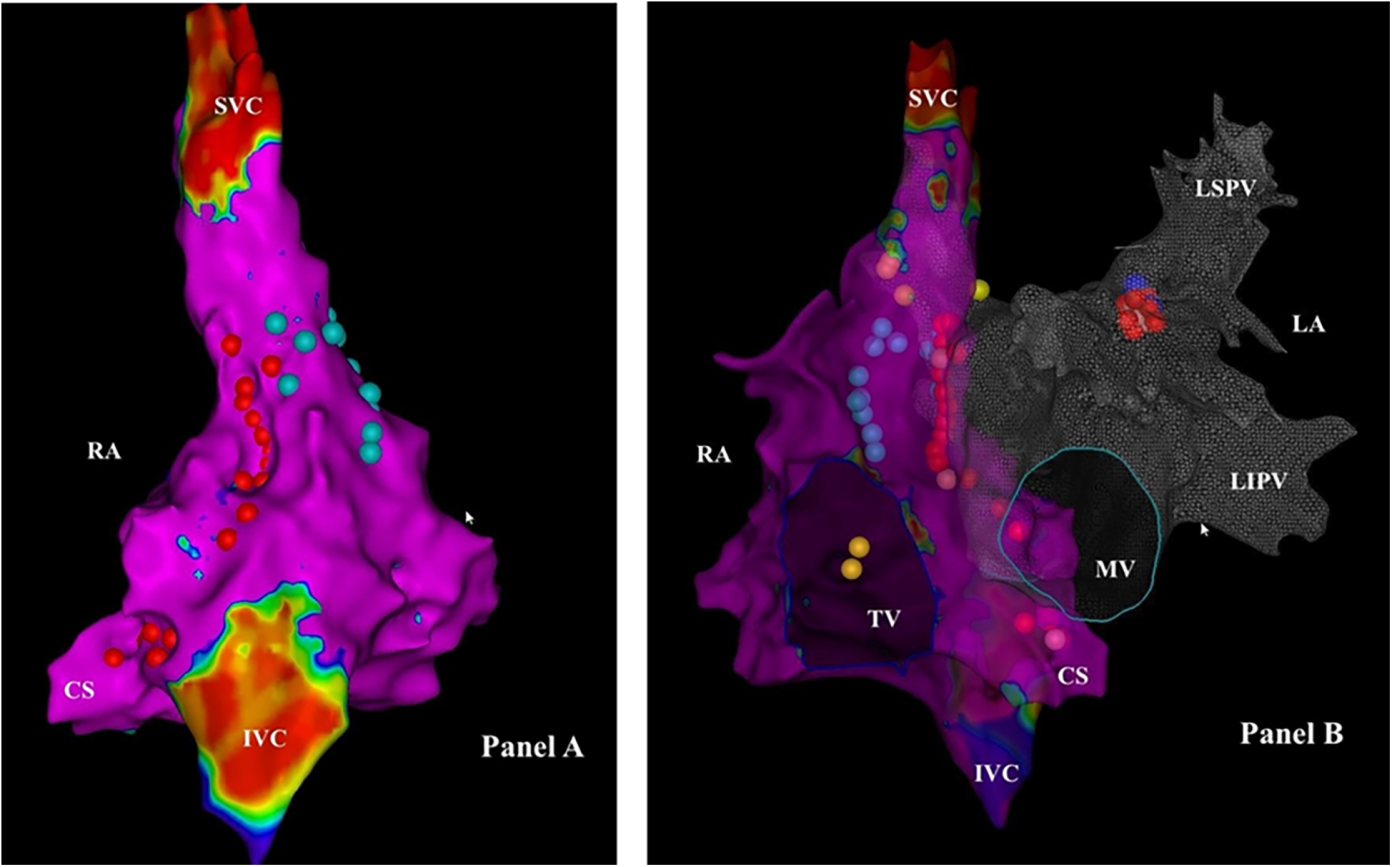
Electroanatomical mapping of the right and left atrium using the CARTO-3 system (biosense webster) in a patient suffering from cardioinhibitory VVS (VASIS 2B), undergoing biatrial. CNA. Panel **(A)** Postero-anterior view of the right atrium - Voltage map. The red dots represent the ablation points in the area of the SPSGP (top) and the IPSGP (bottom). The light blue dots represent the course of the phrenic nerve, highlighted by pacing. Panel **(B)** Left anterior oblique view - Voltage map of the right atrium and Anatomical map of the left atrium. The ablation points in the LSGP area are added to the points in **(A)** The blue dots represent areas where high frequency stimulation resulted in a vagal response. The yellow dots represent the location of the His bundle. CS, coronary sinus; IVC, inferior vena cava; LA, left atrium; LIPV, left inferior pulmonary vein; LSPV, left superior pulmonary vein; MV, mitral valve; RA, right atrium; SVC, superior vena cava; TV, tricuspid valve.

**Figure 2:**
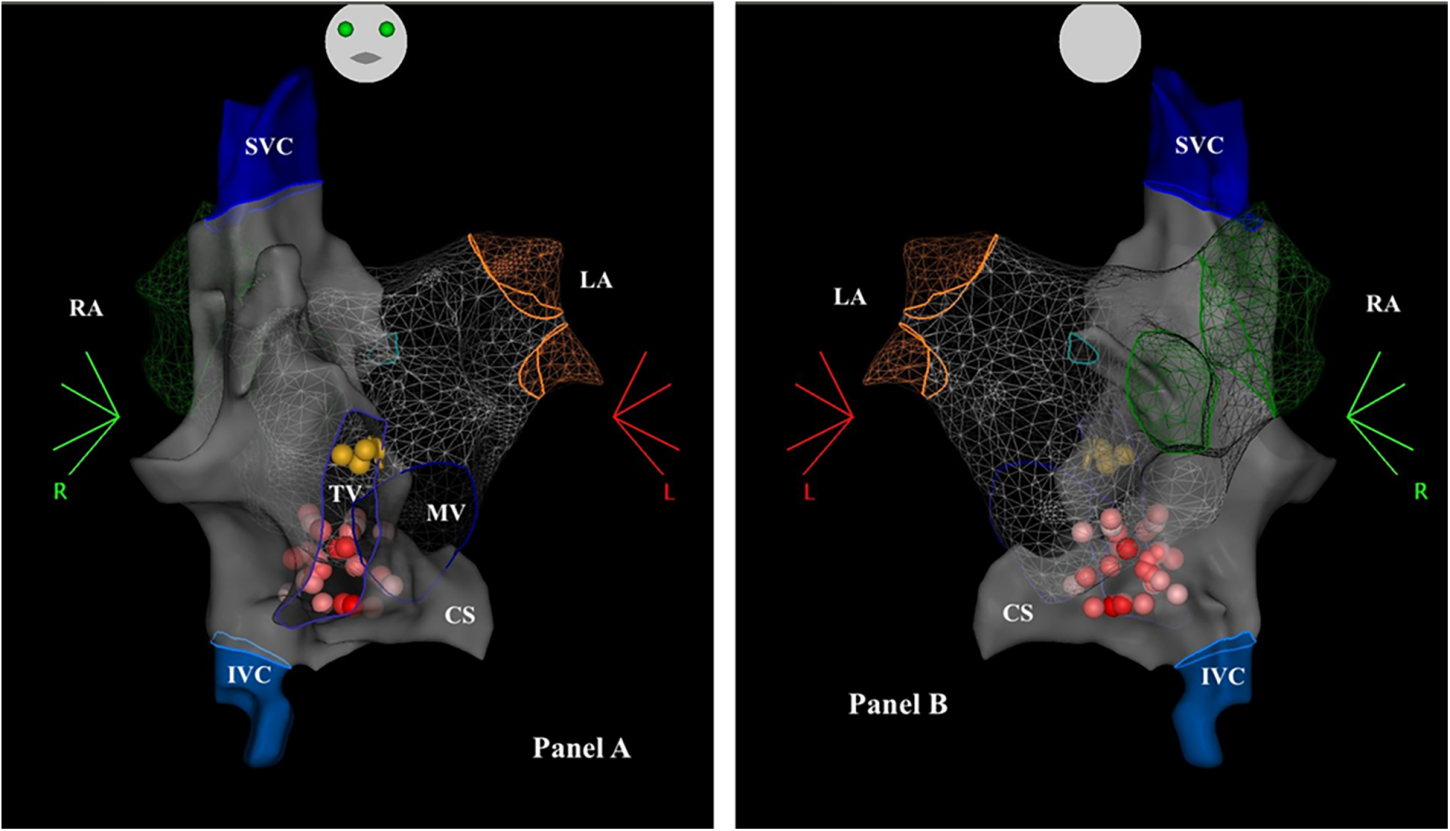
Electroanatomical mapping of the right and left atrium using the CARTO-3 system (biosense webster) in a patient T suffering from functional AV block, undergoing biatrial CNA. Panel **(A)** Antero-posterior view of the atria - Anatomical map. Panel **(B)** Postero-anterior projection - Anatomical map of the atria. The red and the pink dots represent the ablation points with different ablation index, in the area of the IPSGP (from the right and the left side). The yellow dots represent the location of the His bundle. CS, coronary sinus; IVC, inferior vena cava; LA, left atrium; MV, mitral valve; RA, right atrium; SVC, superior vena cava; TV, tricuspid valve.

Some images of our procedures are shown in Figures 1, 2.

### Acute results

**Figure 3:**
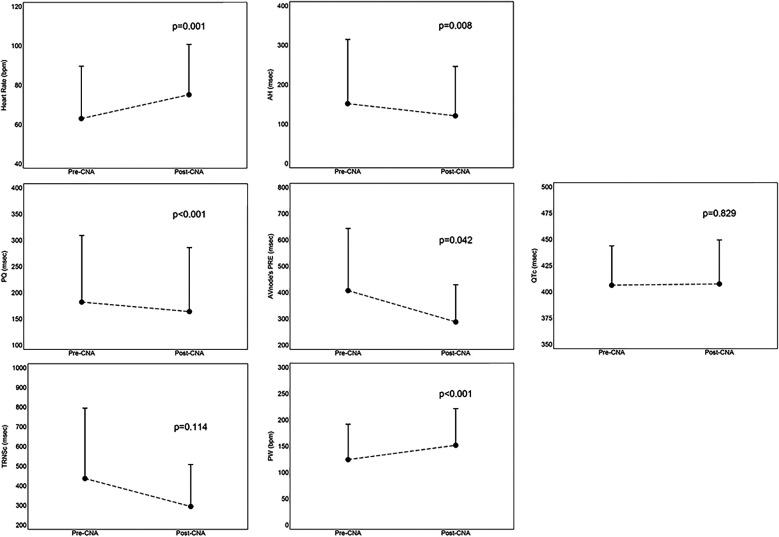
Mean values with standard deviation of acute results, derived from the comparison between parameters assessed before and immediately after the procedure. AH, AH interval; CNA, cardioneuroablation; cSNRT, correct sinus node recovery times; ERP, effective refractory period; PQ, PQ interval; PRE,; PW, point of Wenckebach; QTc, correct QT interval.

The acute results are shown in Figure 3. As regards the acute results of the procedure, we highlighted an increase in sinus heart rate (12 ± 15 bpm, *p* = 0.001), a shortening of the PQ interval (−18 ± 18 msec, *p* < 0.001), a reduction of the cSNRT (−142 ± 204 msec, *p* = 0.114), a shortening of the AH interval (−31 ± 26 msec, *p* = 0.008), a reduction of the effective refractory period of the atrio-ventricular node (−156; interquartile range from −30 to −160 msec, *p* = 0.042) and an increase in the Point of Wencheback (27 ± 20 bpm, *p* < 0.001). The patient who had a fixed third-degree atrio-ventricular block (AVB) upon entering the electrophysiology room, at the end of the procedure had a 1:1 atrio-ventricular conduction, with persistence of only a first-degree AVB (PQ 260 msec). No significant changes in the HV interval were highlighted compared to the pre-CNA values (most of patients had no change; *p* = 0.482). There were no changes in the corrected QT (QTc) interval (1 ± 26 msec, *p* = 0.829), nor development of arrhythmias.

Two patients had a minimal pericardial effusion at pre-discharge echocardiographic control, which did not require any therapeutic intervention; one of these patients underwent ablation of atrial fibrillation and flutter, as well as CNA. No further procedural or post-procedural complications occurred.

In predischarge, among the 8 patients diagnosed with cardioinhibitory VVS, 7 repeated the HUTT: in 3 cases the test was negative for vasovagal syncope, in 2 cases it was conclusive for vasodepressive VVS (VASIS 3) and in 1 case for mixed VVS (VASIS 1). In a single case the HUTT remained positive for cardioinhibitory VVS (VASIS 2B); however, after 12 months of follow-up the patient did not experience syncopal recurrences. The patient with pre-procedure HUTT positive for mixed VVS (VASIS 1) and documentation of asystolic pauses on the loop recorder registrations, had a vasodepressive VVS (VASIS 3) at the post-procedure HUUT.

### Results at follow-up

**Figure 4:**
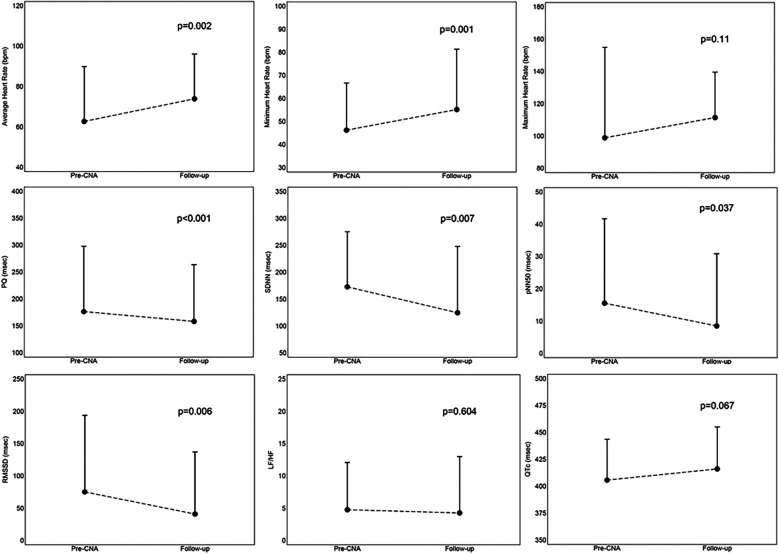
Mean values with standard deviation of results at follow-up, derived from the comparison between parameters assessed before the procedure and at the 12-month follow-up. CNA, cardioneuroablation; LH/HF, ratio between low frequency and high frequency, parameter of frequency domain at Heart Rate Variability; pNN50, percentage of differences between consecutive RR intervals >50 msec, parameter of time domain at Heart Rate Variability; PQ, PQ interval; QTc, correct QT interval; RMSSD, root mean square of the differences between successive normal heart beats, parameter of time domain at Heart Rate Variability; SDNN, standard deviation of all intervals from normal to normal, parameter of time domain at Heart Rate Variability.

The results at follow-up are reported in Figure 4. A single patient, due to persistence of symptoms and bradyarrhythmic disorder (Mobitz 2 s degree AVB) underwent, during a subsequent hospitalization, definitive pacemaker implantation; this was the patient (79 years old) with a history of surgical correction of a sinus venosus type ASD, also subjected to ablation of typical atrial flutter. All remaining patients maintained regular clinical follow-up. During the follow-up no patient had a recurrence of syncope, and they all remained persistently asymptomatic.

We documented a persistence of the increase in the minimum (9 ± 10 bpm, *p* = 0.001) and average (11 ± 14 bpm, *p* = 0.002) heart rate, a shortening of the PQ interval (−18 ± 16 msec, *p* < 0.001), a reduction in time domain (SDNN: −48 ± 62, *p* = 0.007; pNN50: −7 ± 12, *p* = 0.037; RMS SD: −34 ± 43, *p* = 0.006) and frequency domain (LF/HF: −0.5 ± 3.86, *p* = 0.604) parameters in the analysis of heart rate variability (HRV) on the ECG-Holter monitoring performed at follow-up. An increasing trend was also highlighted for the maximum heart rate, although not significant (13 ± 33 bpm, *p* = 0.110); however, no patient experienced palpitations, nor reported discomfort in feeling a higher heart rate, therefore, no patient was pharmacologically treated. There were no significant changes in the QTc interval (10 ± 24 msec, *p* = 0.067) and no patient developed arrhythmic episodes.

**Figure 5:**
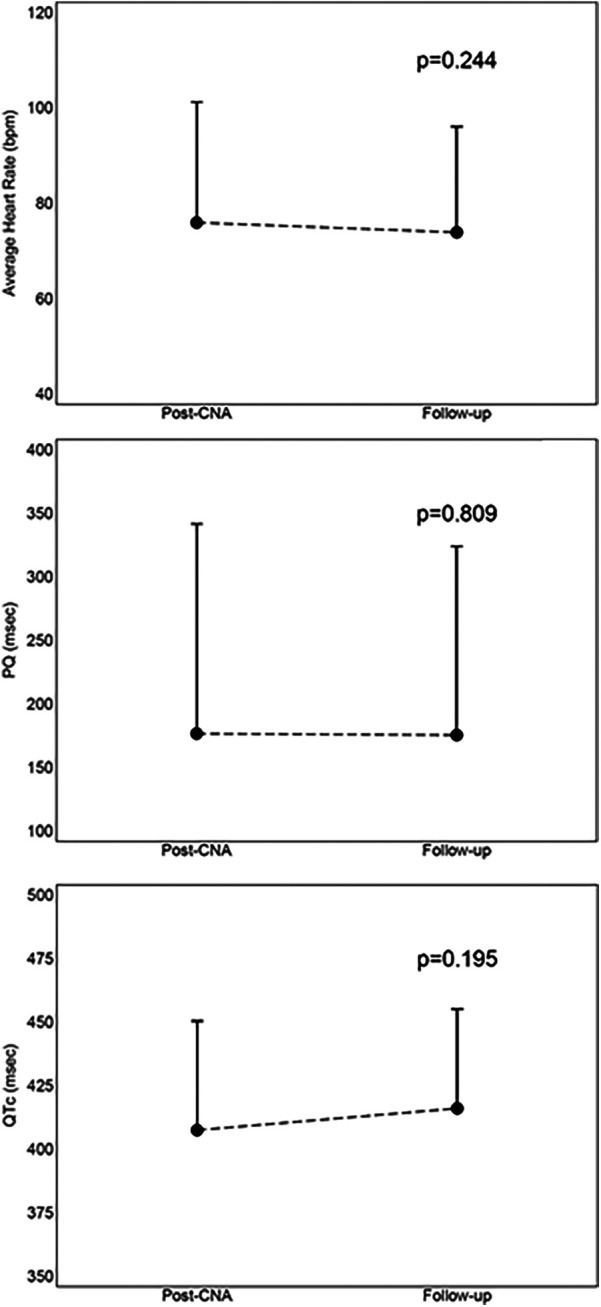
Mean values with standard deviation of comparison between immediately post-procedure results and 12-month follow-up results. CNA, cardioneuroablation; PQ, PQ interval; QTc, correct QT interval.

There were no significant differences between the values of mean heart rate (−2 ± 8 bpm, *p* = 0.244), PQ interval (−1 ± 22, *p* = 0.809) and QTc interval (+9 ± 28, *p* = 0.195) between those measured immediately post-procedure and those measured at follow-up. This comparison is shown in Figure 5.

## Discussion

Cardioneuroablation is a relatively recent and still developing technique, which targets persistent cardiac vagal denervation through endocardial ablation of parasympathetic ganglia. In our prospective non-randomized study on patients suffering from recurrent episodes of VVS (cardioinhibitory or mixed) or symptomatic functional bradyarrhythmias, CNA was effective in reducing the syncopal burden and the other symptoms complained by patients before the procedure.

In the acute phase, statistically significant changes were highlighted in the following parameters: increase in sinus heart rate, shortening of the PQ interval, shortening of the AH interval, reduction of the ERP of the AV node, increase in PW; furthermore, although not statistically significant, we highlighted a decreasing trend in cSNRT. No significant changes in the HV interval were highlighted compared to pre-CNA values.

Except for a single patient who underwent pacemaker implantation due to persistence of lipothymic symptoms and bradyarrhythmic disorder, the rest of the patients had no syncope recurrence and all reported being persistently asymptomatic at follow-up. Therefore, CNA in our series had a success of 95% in terms of absence of syncopal or bradyarrythmias recurrence. Furthermore, during follow-up, statistically significant changes were highlighted in the following parameters: persistent increase in minimum and average heart rate, persistent shortening of the PQ interval; a decreasing trend was also found in the time and frequency domain parameters in the HRV analysis on Holter monitoring, although among these only the time domain parameters were significantly reduced. The increasing trend in maximum heart rate, not statistically significant, probably represents a positive result, since none of our patients complained of symptomatic inappropriate sinus tachycardia, and therefore none required bradycardic therapy.

Regarding to safety, we did not highlight any significant changes in the QTc interval or development of arrhythmias, either in the post-procedural phase or at follow-up. Furthermore, apart from two cases of minimal pericardial effusion which did not require any therapy, we have documented no further procedural complications, and the rate of exposure to ionizing radiation was very low (11 procedures performed with Zero-Fluoroscopy technique, fluoroscopy time average of 2,3 min among the other 10 patients).

The values of the average heart rate, PQ and QTc interval remained stable between the post-procedural phase and the follow-up, demonstrating the persistence of the effect of the CNA.

Adequate selection of patients who could benefit from the neuromodulation procedure is essential. In fact, as regards VVS, since the CNA procedure acts mainly on the cardioinhibitory component of the Bezold-Jarisch reflex, patients with cardioinhibitory VVS (VASIS 2B) at the HUTT should be selected. In our study we mainly selected patients with VVS VASIS 2B, and the post-procedure HUTT evaluations highlighted a prevalent action of the CNA on the cardioinhibitory component. As regards the mixed and vasodepressive forms of VVS, the probability that CNA is effective is reduced, although there is recent evidence relating to the fact that the modulation of one of the reflex components (the cardioinhibitory one) can still be useful for reducing the syncopal burden of patients with non-cardioinhibitory forms of VVS. Even in pure vasodepressive and in mixed forms of VVS, bradycardia contributes to the fall in cardiac output and blood pressure, therefore increasing the heart rate through CNA or pacing could be useful to limit the vasovagal effect also in these forms, especially considering the fact that accurate criteria are not always used for the classification of syncope (2), and that the HUTT has poor reproducibility (12, 16). Indeed, the current clinical results of CNA cannot be explained solely by the effect on cardioinhibition, and it is plausible that CNA influences cardiovascular reflexes in a more global manner (24, 25). In our series we also performed the procedure on a patient with a pre-procedure HUTT positive for mixed VVS (VASIS 1), the post-procedure HUTT remained positive for vasodepressive VVS (VASIS 3) and the patient had no syncopal recurrences at follow-up.

As regards bradyarrhythmias, functional forms should be subjected to CNA, in which the disorder is suspected to derive from hyperactivity of the parasympathetic system (as evidenced by the positive response to Atropine and/or the ergometric test). The probability that the disorder is of an organic type, related to structural damage to the conduction system, increases with age (also because in the elderly there is generally a prevalent sympathetic autonomic regulation), and this is the reason why we tend to subject especially younger patients to CNA. However, there is recent evidence, derived mainly from the ELEGANCE-trial (8), which underlines how age should not in itself be a parameter of exclusion from the execution of the CNA procedure; this study evaluated the possibility of performing the CNA procedure also on elderly patients, demonstrating that, although the increase in heart rate was less significant and less lasting in the group of older patients, there were no significant differences in terms of syncopal recurrences between the different age groups. Obviously, precisely because of the greater probability that the elderly have an organic disorder, a strict and accurate evaluation is necessary before subjecting them to the CNA procedure. In line with these assessments, among our 21 patients only 1 underwent definitive pacemaker implantation due to persistence of symptomatic functional bradyarrhythmia, and he was in fact one of the two older patients (79 years old), previously subjected to cardiac surgery for a sinus-venous type ASD, symptomatic for situational syncopal episodes and with evidence of nocturnal episodes of Mobitz 2 s-degree AVB: probably in this case the bradyarrhythmic disorder had a prevalent organic component. However, the oldest patient in our series (81 years old), symptomatic for dyspnea due to mild exertion and lipothymic episodes, with evidence of nocturnal episodes of Mobitz 2 s-degree II AVB, underwent biatrial CNA in addition to ablation of atrial fibrillation and typical right atrial flutter, had good results and remained asymptomatic at 17 months of follow-up.

As regards procedural aspects, there is still a significant heterogeneity in the CNA protocols between the various groups, especially regarding the methods of localization of the paraganglia and validation of the procedure, as well as regarding the correct extension of the lesions (mono-atrial or bi-atrial). In our case studies, for the localization of the GPs we used the anatomical approach, and only in some cases the HFS; the anatomical approach is based on the assumption that the parasympathetic ganglia are typically located in similar positions in almost all patients, thus allowing an empirical ablation of these regions with high success rates and identical to the other approaches, but with shorter procedure and fluoroscopy times (26). However, it is important to point out that there are other methods for GP localization in addition to those already mentioned: spectral analysis, fractional electrogram research, single photon emission computed tomography (CT) or CT (3, 10, 16, 18, 26–30). Since comparative data between the methods are not available, the optimal GP localization technique to prevent reinnervation and obtain long-term clinical benefits has yet to be determined (31). In this regard, the recent meta-analysis on CNA found no differences in subgroup analysis, comparing different methods of localization of target sites for GP ablation (6). All CNA approaches should be guided by a 3D navigation system and performed using temperature-controlled radiofrequency (RF) delivery, preferably with irrigated catheter up to 30 W/45°C (16). Each RF delivery is interrupted according to the delivery time or the Ablation Index (AI, a parameter that takes into consideration power, time and stability of the lesion performed) (31). Since the average thickness of the atrial wall is approximately 3–4 mm, radiofrequency energy can be transmitted through the atrial wall to the epicardial paraganglia, allowing its ablation through the endocardium (16). We used an AI of 350 for the deliveries in the coronary sinus and 550 for the deliveries on the atrial wall, with the aim of reaching a lesion depth of approximately 8 mm, which allowed to impact on the epicardial GPs. For the choice of GPs to ablate, we based ourselves on the rhythm disturbance highlighted clinically or during HUTT, directing the ablation towards the ganglia probably responsible. For example, for patients with documentation of asystole or severe sinus bradycardia, ablation was directed predominantly to the superior paraseptal ganglionated plexus (SPSGP), the common end station of the parasympathetic innervation of the sinus node (2, 12, 16), with extension to the left superior ganglionated plexus (LSGP) in case of an unsatisfactory result.

As regards patients with documentation of atrioventricular conduction disorders, we mainly targeted the inferior paraseptal ganglionated plexus (IPSGP), the common final station of the parasympathetic innervation of the AV node (2); in such cases we pursued selective denervation of the AV node to avoid excessive acceleration of sinus rhythm, particularly for patients with a high resting sinus rate and/or notable acceleration of sinus rate after atropine administration, for avoid worsening the AV conduction due to the acceleration of the sinus rate. Some evidence supports a more extensive ablation for VVS, because it has been demonstrated that, although in most cases the prevalent bradyarrhythmia is sinus, it is possible that its presence hides underlying AV conduction anomalies (in case of sinus arrest the AVB cannot be observed due to the absence of atrio-ventricular conduction), or that after denervation of the sino-atrial node the pathophysiology changes with syncopal recurrence due to high-grade AV block (24, 32–34). For this reason, in most patients with VVS we performed an ablation extended to the SPSGP, LSGP and IPSGP. Naturally, the “titration” of the ablation is purely empirical, and in general it is more correct to think of an ablation strategy adapted to the individual patient, balancing the benefits and risks of the procedure (35, 36). Regarding the extent of ablation, although neuroanatomical studies on cadavers of people without known cardiovascular disease have demonstrated that the majority of cholinergic fibers access the human heart through the right atrium, supporting the idea that one can reasonably limit the ablation on the right atrium to balance efficacy and safety (2, 37), however, the meta-analysis by Vandenberk et al. highlighted that CNA limited to the right atrium was associated with significantly lower freedom from syncope (81.5%) compared to ablation of the left atrium (94%) and bi-atrial (92.7%) (6). In fact, it has been seen that the greatest density of ganglia that innervate the sinoatrial node is usually found at the junction of the superior vena cava with the right atrium, in the thick superior interatrial septum; this anatomical distribution could be the reason why in most cases biatrial ablation is necessary to achieve complete denervation of the sinoatrial node (2, 16, 18, 33). This is even more true regard to complete denervation of the AV node, since ablation of the IPSGP almost always requires a biatrial approach. In fact, a recent prospective randomized study published on the topic (ROMAN2) demonstrated that the achievement of complete vagal denervation in acute conditions was significantly greater with the left atrial approach compared to the right atrial approach, and that complete vagal denervation of the AV node most frequently requires a bilateral approach (38). Also in our experience, denervation of the AV node has always required a biatrial approach, while as regards denervation of the sinus node a right atrial approach has sometimes been sufficient. Depending on the GP mapping method used, there are several endpoints, useful for procedural validation: elimination of fractionated electrograms, switching off of the vagal response (VR) triggered by ablation or HFS, response to extracardiac vagal stimulation (ECVS, typically performed by the right internal jugular vein near the vagus nerve, to trigger a vagal cardioinhibition reflex through a neurostimulator designed by Dr. Pachon), substantial changes in electrophysiological parameters compared to the pre-procedural phase, changes in the response to atropine administration (2). Again, each method has advantages and disadvantages, and it is not known which procedural endpoint guarantees better long-term results, although the evidence for ECVS is promising. In our case, however, since in Italy there is no availability of devices for extracardiac vagal stimulation (ECVS), we used as a procedural target the persistent modification of electrophysiological parameters such as sinus frequency and the PW (which however represent indirect parameters and not necessarily indicative of a complete effect) (39), and the switching off of the irritative vagal response triggered during ablation (10, 30).

A potential limitation of CNA is the possibility that reinnervation restores vagal hyperactivity. The general concept is that if the cell body of the neuron is damaged the cell will not regenerate, while if only the post-ganglionic fibers are damaged axonal and synaptic regeneration will occur. Therefore, considering the localization of postganglionic parasympathetic neurons at the level of the epicardial GPs or atrial wall, vagal reinnervation is a much rarer event than sympathetic reinnervation, especially if the procedure is performed correctly based on achieving an adequate Ablation Index (2, 5, 26, 31, 37, 40). Furthermore, although some degree of reinnervation may occur, it may be partial and there is insufficient data to indicate that the extent of recovery is associated with the risk of syncope recurrence. Furthermore, the natural history of reflex syncope in young patients is characterized by a high rate of spontaneous remission of syncopal episodes with advancing age. It can therefore be hypothesized that complete and permanent atrial denervation may not be necessary and that, after all, the reinnervation process may not be a bad thing, because the decrease in heart rate and the recovery of its variability could for example help to attenuate post-procedural sinus tachycardia (which some patients present in a symptomatic form) (2, 26, 31, 40).

The placebo effect is also a critical issue when considering the effectiveness of GP ablation. In previous studies on the treatment of VVS with pacemaker implantation, a huge impact of the placebo effect was found. Therefore, although the procedure causes persistent changes in electrophysiological parameters (objective data), in the absence of randomized studies conducted blindly and with a sham procedure for the control group, the existence of an important placebo effect cannot be excluded (5, 10, 16).

### Study limitations

Our study has some limitations: first, it included a small number of patients without a control group (which limits assessments of the potential impact of the placebo effect); we did not perform post-procedural atropine test in all cases; another limitation is represented by the heterogeneity of the methods of localization of the paraganglia used, in some cases the anatomical approach, in others the HFS; finally, the duration of follow-up was relatively short, therefore, our study does not provide sufficient evidence to draw conclusions about the duration of CNA effects and the potential development of long-term vagal denervation side effects.

## Conclusions

Our results support the efficacy and safety of CNA for the treatment of VVS and functional bradyarrhythmias. Patients affected by these conditions manifest recurrent symptoms which negatively impact the quality of life. The therapeutic strategies adopted so far, including pacemaker implantation, are often ineffective, and expose patients to a high risk of long-term complications and further worsen their quality of life. In this context, CNA appears to be a promising treatment, although well-designed randomized controlled trials are needed to further test its efficacy and safety, and standardize the methodic, to evaluate its inclusion in future guidelines with the aim of improving patient care.

## Data Availability

The original contributions presented in the study are included in the article/Supplementary Material, further inquiries can be directed to the corresponding author.
